# LncRNA‐GPHN Regulates Epilepsy by Inhibiting Apoptosis via the miR‐320/YWHAH Axis in an Immature Rat Model of Status Epilepticus

**DOI:** 10.1111/jcmm.70593

**Published:** 2025-05-10

**Authors:** Jun Chen, Xueyi Rao, Xiaoqian Wang, Yang Li, Yajun Shen, Jing Gan

**Affiliations:** ^1^ Department of Pediatrics, West China Second University Hospital Sichuan University Chengdu Sichuan China; ^2^ Ministry of Education Key Laboratory of Birth Defects and Related Diseases of Women and Children (Sichuan University) Chengdu China; ^3^ Department of Pediatrics Meishan City People's Hospital Meishan Sichuan China; ^4^ WCSUH‐Tianfu·Sichuan Provincial Children's Hospital Meishan City Sichuan Province China

**Keywords:** epilepsy, GPHN, lncRNA, miR‐320, YWHAH

## Abstract

Long non‐coding RNAs (lncRNAs) play significant roles in neurological diseases, including epilepsy. Our previous study identified lncRNA‐GPHN as specifically downregulated in a rat model of status epilepticus (SE). Investigate the role and regulatory mechanism of lncRNA‐GPHN in the pathogenesis of epilepsy. SE rat and in vitro cell models were used to analyse expression dynamics, cellular localisation, and effects of lncRNA‐GPHN on epileptic seizures, followed by HE staining, Nissl staining, and TUNEL staining. Luciferase Reporter assay, ChIRP assay, real‐time quantitative PCR, and Western blotting accompanied with TUNEL assay and whole‐cell patch‐clamp techniques were employed to determine the molecular mechanism in lncRNA‐GPHN regulating epilepsy in neurons. Post‐seizure, lncRNA‐GPHN in SE rats' hippocampus was markedly downregulated, hitting a nadir at 24 h. FISH and qPCR confirmed its cytoplasmic localization in neurons. EEG showed that lncRNA‐GPHN overexpression significantly curtailed seizure frequency and intensity, elevating the threshold, while MWM results pointed to enhanced cognition in SE rats. Histological staining revealed less neuronal damage and better cellular integrity in overexpressing rats, accompanied with a reduction in neuronal apoptosis. In vitro, lncRNA‐GPHN reduced neuronal excitability and epileptic potentials dose‐dependently. q‐PCR and ChIRP showed lncRNA‐GPHN upregulates YWHAH by sequestering miR‐320. Dual‐luciferase and Western blot validated miR‐320's direct suppression of YWHAH and lncRNA‐GPHN's counteracting effect. TUNEL staining confirmed that miR‐320 overexpression increased apoptosis, mitigated by lncRNA‐GPHN overexpression and further reduced with combined overexpression. lncRNA‐GPHN ameliorates epilepsy by inhibiting apoptosis via the miR‐320/YWHAH axis, providing insights into epilepsy pathogenesis and potential targeted therapeutic strategies.

## 1 Introduction

Epilepsy is a chronic neurological disorder characterised by recurrent seizures due to abnormal electrical discharges in the brain. Approximately 50 million people worldwide suffer from epilepsy, making it one of the most common serious neurological diseases [1]. Epilepsy not only imposes physical and psychological burdens on patients but also significantly impacts society and healthcare systems [2]. Despite significant advances in epilepsy treatment over the past decades, about 30% of epilepsy patients remain resistant to existing medications [3], indicating an urgent need for a deeper understanding of epilepsy pathogenesis to develop new therapeutic strategies.

Long noncoding RNAs (lncRNAs), a class of noncoding RNA molecules longer than 200 nucleotides, about 40% of lncRNAs are expressed in the brain, playing important roles in neural development, neural plasticity, cognitive function, and synaptic plasticity [4, 5]. Increasing evidence suggests that lncRNAs play key roles in various neurological diseases [6], by regulating the expression of downstream genes through interactions with miRNAs [7]. For instance, in Alzheimer's disease (AD), the lncRNA NEAT1 can bind to miR‐124 and negatively regulate the expression of beta‐site amyloid precursor protein cleaving enzyme 1 (BACE1), playing a crucial role in AD development [8]. Furthermore, lncRNA‐NEAT1 can directly bind and negatively regulate miR‐107 expression, exacerbating beta‐amyloid‐induced neuronal damage, and offering new avenues for AD treatment [9]. Additionally, lncRNA‐NEAT1 participates in the development of central nervous system diseases such as Parkinson's disease (PD), Huntington's disease (HD), and amyotrophic lateral sclerosis (ALS) through various pathways, including the regulation of autophagy, apoptosis, and neuroinflammation [10, 11, 12].

Recent studies have shown that lncRNAs may play significant roles in the molecular pathways and cellular processes involved in epilepsy, such as neuroinflammation, cell proliferation and differentiation, migration, apoptosis, and synaptic remodelling [13, 14]. Therefore, studying the role of lncRNAs in the pathogenesis of epilepsy is crucial for identifying new therapeutic targets. Based on previous microarray results [15], we identified a lncRNA specifically downregulated in epilepsy: IncRNA‐NONRATTO25141.2, which is located in the first intron of the Gephyrin (GPHN) gene on rat chromosome 6. Therefore, we named it IncRNA‐GPHN, but its specific role and regulatory mechanism in epilepsy pathogenesis remain unclear. This study aims to investigate the role of lncRNA‐GPHN in the pathogenesis of epilepsy by establishing an epilepsy rat model and an in vitro epilepsy cell model, thereby providing a theoretical foundation for developing novel anti‐epileptic therapeutic strategies targeting lncRNA‐GPHN.

## 2 Materials and Methods

### 2.1 Experimental Animals

All animal experiments were approved by the Sichuan University Committee on Animal Research. Male Sprague–Dawley rats weighing 50–60 g were obtained from Chengdu Dashuo Laboratory Animal Co. Ltd. (Chengdu, China). The rats were housed in a temperature‐controlled room (22°C ± 2°C) with a 12‐h light/dark cycle and had free access to standard food and water. All procedures were conducted in accordance with the Chinese Animal Welfare Act and the guidelines of the Sichuan University Experimental Animal Center. The study was approved by the Ethics Committee of West China Second Hospital, Sichuan University.

### 2.2 Status Epilepticus Model

Status epilepticus (SE) was induced using a lithium chloride–pilocarpine protocol. Rats received an intraperitoneal injection of lithium chloride (125 mg/kg, Sigma) 18–20 h before the induction of seizures. Thirty minutes prior to pilocarpine administration (35 mg/kg, i.p., Sigma), rats were given scopolamine (1 mg/kg, i.p., Sigma) to mitigate peripheral cholinergic effects [16]. Seizures were graded according to the Racine scale (Table 1) and were manually scored by two blinded researchers to ensure objective evaluation. Rats reaching stage 4 or 5 were considered to have successfully developed SE. Fifty minutes after SE onset, seizures were terminated with 10% chloral hydrate (3 mL/kg, i.p., Sigma). Rats failing to reach stage 4 or 5 following two injections of pilocarpine (10 mg/kg, i.p.) and a subsequent 10‐min observation were euthanized and excluded from the study.

**TABLE 1:** Racine scale.

Score	Behavioural stage
0	No change in behaviour
1	Sudden behaviour arrest, motionless staring (with orofacial automatism)
2	Head nodding
3	Forelimb clonus with lordotic posture
4	Forelimb clonus with rearing and falling
5	Generalised tonic–clonic activity with loss of postural tone, wild jumping

### 2.3 Construction and Overexpression of GPHN in Rats

Adeno‐associated viral (AAV) injections (6.36E +13v.g per mL) were administered using an AAV carrying GPHN (lncRNA NONRATT025141.2) or a control vector (AAV‐NC), constructed by GenPharma (Shanghai, China). One day after establishing the epilepsy model, SE rats were randomly divided into two groups. The rats were anaesthetised with 10% chloral hydrate (3 mL/kg, i.p., Sigma) and fixed in a prone position. A midline incision was made on the front edge of the ear line to expose the anterior fontanel. A total of 5 μL AAV vectors were injected into the left lateral ventricle through a microsyringe at 0.5 μL/min (coordinates: AP = −0.8 mm, ML = −1.2 mm, and DV = −2.6 mm from the skull surface). The needle was kept in place for 3 min before removal, and the skin was sutured.

### 2.4 Epileptic Cellular Model in Primary Hippocampal Neurons

Primary hippocampal neurons were isolated from 24‐h‐old SD rats. Hippocampi were extracted, cut into small pieces, and digested with 0.2% trypsin at 37°C until dissociated into a cell suspension. The digestion was stopped, and cells were suspended in DMEM with 10% FBS and cultured under humidified conditions (37°C, 5% CO2). After 4 h, the medium was replaced with complete neurobasal medium. Immunofluorescence was used to identify the isolated hippocampal neurons. After 10 days of culture, neurons were immersed in magnesium‐free extracellular fluid (composition: 137 mmol/L NaCl, 4 mmol/L KCl, 1.8 mmol/L CaCl2, 10 mmol/L HEPES, 10 mmol/L glucose, pH adjusted to 7.4, osmolarity adjusted to 280–320 mOsm with sucrose) to test cell excitability.

### 2.5 Electroencephalogram (EEG)

Seven days after AAV injections, rats were anaesthetised with chloral hydrate (10%, 3 mL/kg, i.p.), fixed on a stereotaxic apparatus, and implanted with three silver electrodes over the frontal and bilateral parietal cortices (BL‐420F system, Chengdu Taimeng Software Co. Ltd., China). Dental cement was used for fixation. Data were collected at 500 Hz and bandpass filtered at 50 Hz for 1 h. The EEG recordings were visually examined by two researchers unaware of the grouping. Epileptiform EEG was defined as a spike–wave pattern with a frequency of 4–8 Hz, an amplitude of about 1.5–2 times the baseline, and lasting over 1 s.

### 2.6 Pentylenetetrazol (PTZ) Seizure Induction

Seven days after AAV injection, rats were administered PTZ (10 mg/kg, i.p., Sigma) every 5 min. Latencies to the first myoclonic jerk (MJ) and generalised tonic–clonic seizure (GTCS) were recorded by two researchers unaware of the grouping.

### 2.7 Morris Water Maze (MWM)

To evaluate spatial learning and memory ability, the MWM was conducted 7 days after lateral ventricle injection. Rats (*n* = 8) were taken to the testing room and kept in single cages 30 min before the experiment. The experiment was conducted in a circular pool (diameter: 150 cm; height: 50 cm) with black water at 22°C–24°C. The pool was divided into four quadrants with few visual cues. A black escape platform (13 × 13 cm) was placed 1 cm below the water surface in the target quadrant. Rats were released randomly from four quadrants and trained to find the hidden platform for 5 consecutive days. If a rat failed to find the platform within 1 min, it was guided to the platform and stayed for 15 s. The interval between consecutive trials was 30 min. A probe trial was conducted 1 day after the training trial ended. The platform was removed, and the rats were placed in the opposite quadrant to search for the platform for 60 s. Escape latency and the number of crossings were recorded using a video‐tracking system (Smart 3.0 Premium, Harvard/Panlab, USA).

### 2.8 Haematoxylin and Eosin (HE) Staining

Seven days after the AAV injection, rats were anaesthetised with 10% chloral hydrate (3 mL/kg) and perfused intracardially with 50 mL sterile saline, followed by 50 mL of 4% paraformaldehyde. Brain tissues were fixed overnight at 4°C in 4% paraformaldehyde. Paraffin‐embedded brain sections (5 μm) were prepared and stained with HE staining solution. Pathological changes were examined at 400x magnification using an Olympus optical microscope (Olympus Optical Co. Ltd., Tokyo, Japan) and analysed with ImageJ software.

### 2.9 Nissl Staining

Seven days after the injection of AAV in rats, the brain tissues were collected, embedded in paraffin, dewaxed, rehydrated, and immersed in 1% toluidine blue for 15 min. Sections (10 μm) were prepared for routine observation. After washing in distilled water, sections were dehydrated in graded ethanol (70%, 85%, 90%, 95%, and 100%), cleared in xylene, and cover‐slipped with neutral balsam. The number of surviving neurons in CA1 and CA3 was analysed using ImageJ software [17]. Six sections per brain were assessed blindly.

### 2.10 Terminal Deoxynucleotidyl Transferase Mediated dUTP Nick‐End Labeling (TUNEL) Assay

Samples were deparaffinised, hydrated in gradient ethanol, and treated with 20 mg/L protease K for 15 min at room temperature. Sections were then incubated with 50 μL TdT labeling buffer containing TdT enzyme (1:9) for 60 min at 37°C. After staining with DAPI for 5–10 min and washing with PBS, slides were sealed with an antifluorescence quencher. TUNEL‐positive cells were observed and analysed using a fluorescence microscope (Olympus Co., Japan).

### 2.11 Cell Culture and Treatment

293T cells were cultured in DMEM with 10% FBS (MilliporeSigma) and 1% penicillin/streptomycin. Cells were purchased from the American Type Culture Collection and confirmed to be mycoplasma‐free using a detection kit (Yise Medical Technology). Cells were maintained at 37°C and 5% CO_2_. Overexpression plasmid of GPHN and miR‐320 mimic were purchased from Genepharma and RiboBio, respectively.

### 2.12 Nucleocytoplasmic Separation Assay

Nuclear and cytoplasmic protein components were extracted in accordance with the kit's NE‐PER Nuclear and Cytoplasmic Extractant (Thermo Scientific) product instruction. RNA purity was validated by measuring nuclear marker U6 snRNA and cytoplasmic marker GAPDH mRNA. The isolated RNA was reverse transcribed and quantified by qPCR.

### 2.13 RNA Extraction and Quantification qPCR

RNA was extracted from tissues and cell lines using TRIzol reagent (Thermo Fisher Scientific). RNA purity and concentration were assessed using a Nanodrop 2000 UV–Vis spectrophotometer. One microgram of RNA was reverse‐transcribed into cDNA using the PrimeScript RT Reagent Kit. qPCR was performed using SYBR Green Master Mix on an ABI 7500 Fast Real‐Time PCR System (Applied Biosystems) with the following conditions: 95°C for 600 s, followed by 42 cycles of 95°C for 5 s and 58°C for 30 s. Expression levels of GAPDH, β‐actin, and U6 snRNA were used as internal controls. Relative gene expression levels were calculated using the $2^{-\Delta \Delta C_{t}}$ method.

### 2.14 Whole‐Cell Patch Clamp Recordings

Whole‐cell recordings were performed using Patchmaster software. Data processing and current traces were analysed using Igor Pro and GraphPad Prism 7.0. Results are presented as mean ± SD. Statistical comparisons were made using one‐way ANOVA.

### 2.15 Western Blotting

Cells and tissues were lysed using RIPA buffer (Beyotime) with protease and phosphatase inhibitors (Roche). Protein concentrations were determined using a BCA assay kit (Thermo Fisher Scientific). Ten micrograms of total protein lysates were separated by SDS‐PAGE and transferred onto PVDF membranes (Invitrogen) at 100 V for 60 min. Membranes were blocked with 5% nonfat milk for 2 h and incubated overnight with anti‐GAPDH (1:10000; ABclonal, AC002) at 4°C. After washing, membranes were incubated with HRP‐conjugated secondary antibodies (1:5000; ZSGB‐BIP). Bands were visualised using ECL substrate (Thermo Fisher Scientific) and images captured with the iBright CL1000 Instrument (Invitrogen).

### 2.16 Fluorescence In Situ Hybridisation (FISH)

Cells were fixed with 4% paraformaldehyde for 10 min at room temperature, permeabilized with 0.5% Triton X‐100 for 5 min at 4°C, and blocked with pre‐hybridisation buffer for 30 min at 37°C. Cells were incubated with FISH probe hybridisation buffer overnight at 37°C in the dark. Nuclei were stained with DAPI. Images were captured using a fluorescence microscope. FISH probes and kits were obtained from RiboBio (Guangzhou).

### 2.17 Chromatin Isolation by RNA Purification (ChIRP)

Cells were crosslinked with 1% glutaraldehyde and lysed with lysis buffer. Chromatin was sheared to 100–500 bp fragments using ultrasound. Lysates were incubated with biotin‐labelled probes from RiboBio at 37°C for 4 h. Magnetic beads were added to separate the probes. RNA fragments were washed, purified, and quantified by qPCR.

### 2.18 Luciferase Reporter Assay

Luciferase reporter vectors containing wild‐type (WT) and mutant (Mut) YWHAH‐3′UTR binding sites for miR‐320 were constructed. 293T cells were seeded in six‐well plates and co‐transfected with luciferase reporter vectors and miRNA expression plasmids using Lipofectamine 3000 (Thermo Fisher Scientific). After 48 h, cell lysates were prepared and luciferase activity measured using the GeneCopoeia dual‐luciferase reporter assay system.

### 2.19 Bioinformatics Analysis

miRbase (http://www.mirbase.org) and TargetScan (www.targetscan.org) were used to detect the potential targets of miRNA. The sequences of rat miRNA were retrieved from miRBase (http://www.mirbase.org/). The binding sites of miRNA to YWHAH were predicted using the web‐based program TargetScan.

### 2.20 Statistical Analysis

Data were analysed using GraphPad Prism 7.0 and presented as mean ± SD. Normality was assessed using the Shapiro–Wilk test. For normally distributed data, statistical significance was determined using unpaired or paired Student's t‐test, one‐way ANOVA, or two‐way ANOVA as appropriate. Sample sizes and the number of experimental repetitions are provided in the figure legends. A *p*‐value < 0.05 was considered statistically significant.

## 3 Results

### 3.1 Expression and Distribution of lncRNA‐GPHN in SE Rat Brain Tissue

**FIGURE 1:**
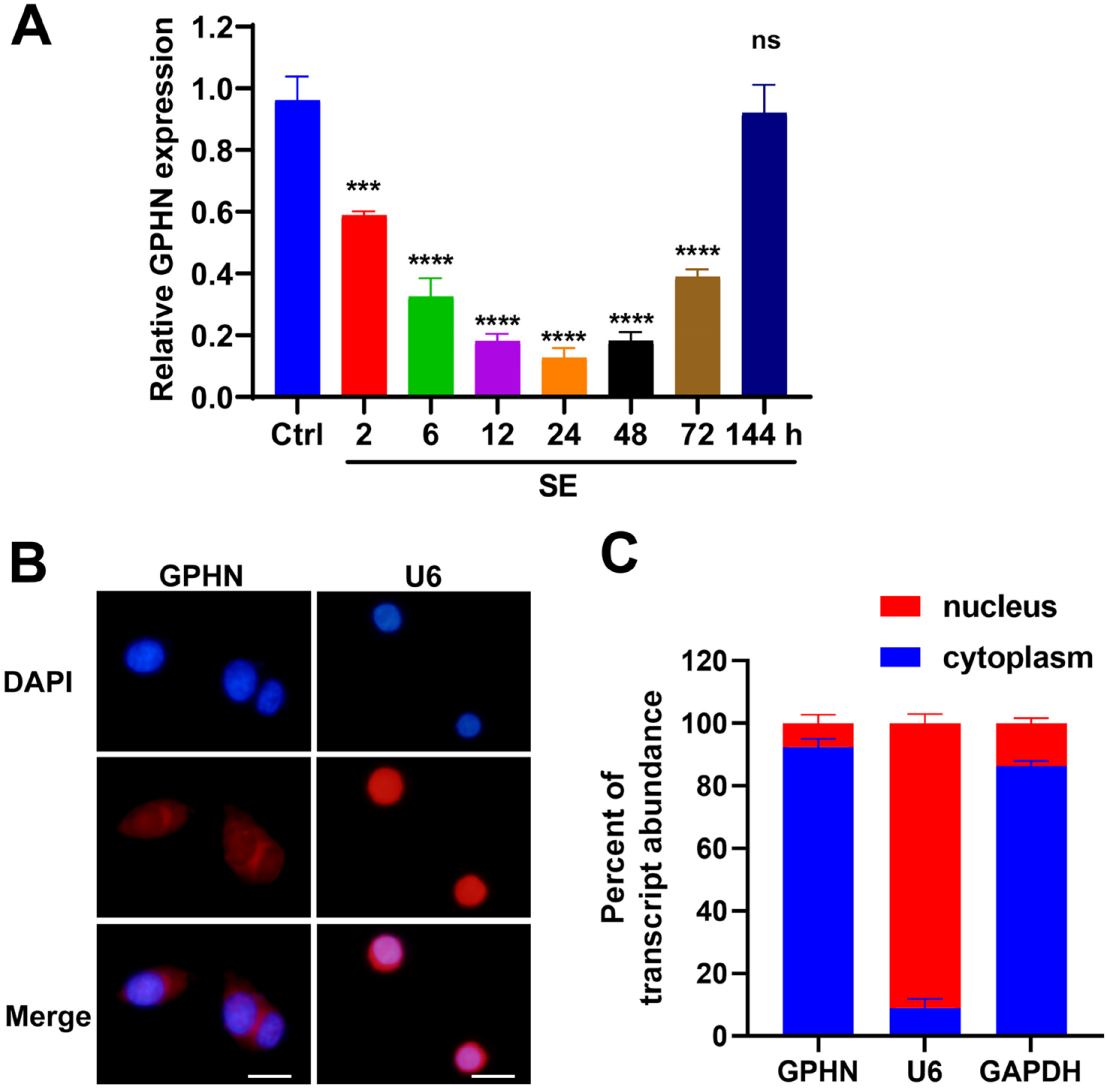
Expression and distribution of lncRNA‐GPHN in rats after SE onset. (A) Real‐time qPCR was used to detect lncRNA‐GPHN expression levels in the hippocampal tissue of SE rats. Results showed that lncRNA‐GPHN expression significantly decreased immediately after seizures, continued to decline at 6 and 12 h, reached its lowest point at 24 h, and then gradually returned to near‐baseline levels at 48 and 72 h, fully recovering to baseline levels by 144 h (7 days) (*n* = 3; ***p < 0.001, ****p < 0.0001, compared to the control group). (B) FISH staining was used to detect the subcellular localization of lncRNA‐GPHN, showing that lncRNA‐GPHN is mainly localised in the cytoplasm (red). Scale bar = 15 μm. (C) Nuclear–cytoplasmic fractionation qPCR in 293 T cells. Purity of the separation was validated by U6 snRNA enrichment in the nuclear fraction (91.1% ± 5.1%) and GAPDH mRNA enrichment in the cytoplasmic fraction (86.2% ± 2.9%). Quantification of lncRNA‐GPHN revealed predominant cytoplasmic localization (92.3% ± 4.8% cytoplasmic vs. 7.7% ± 4.8% nuclear; *p* < 0.001), in agreement with FISH analysis.

To investigate the expression of lncRNA‐GPHN in SE, we used real‐time qPCR to detect its expression levels in the hippocampal tissue of SE rats. The results showed that lncRNA‐GPHN expression significantly decreased immediately after seizures (*p* < 0.01), continued to decline at 6 and 12 h, reached the lowest point at 24 h (*p* < 0.001), and then gradually returned to near‐baseline levels at 48 and 72 h, reaching baseline levels by 144 h (7 days) (Figure 1A). This indicates that lncRNA‐GPHN expression significantly decreases immediately after seizures, reaching its lowest point at 24 h.

To further clarify the localisation of lncRNA‐GPHN, we used FISH staining and nuclear‐cytoplasmic separation‐quantitative PCR to examine its expression distribution in immature rat brain tissue. In FISH staining, DAPI was used to label nuclei, and by comparing lncRNA‐GPHN to U6 snRNA as a nuclear reference control, we found that lncRNA‐GPHN is mainly localised in the cytoplasm (Figure 1B). The nuclear‐cytoplasmic fractionation qPCR results demonstrated that lncRNA‐GPHN was predominantly cytoplasmic, and the purity of the separation was validated by the nuclear enrichment of U6 and cytoplasmic enrichment of GAPDH (Figure 1C). These findings consistently indicate that lncRNA‐GPHN is primarily localised in the neuronal cytoplasm.

### 3.2 lncRNA‐GPHN Improves Epileptic Discharges and Cognitive Impairment in SE Rats

**FIGURE 2:**
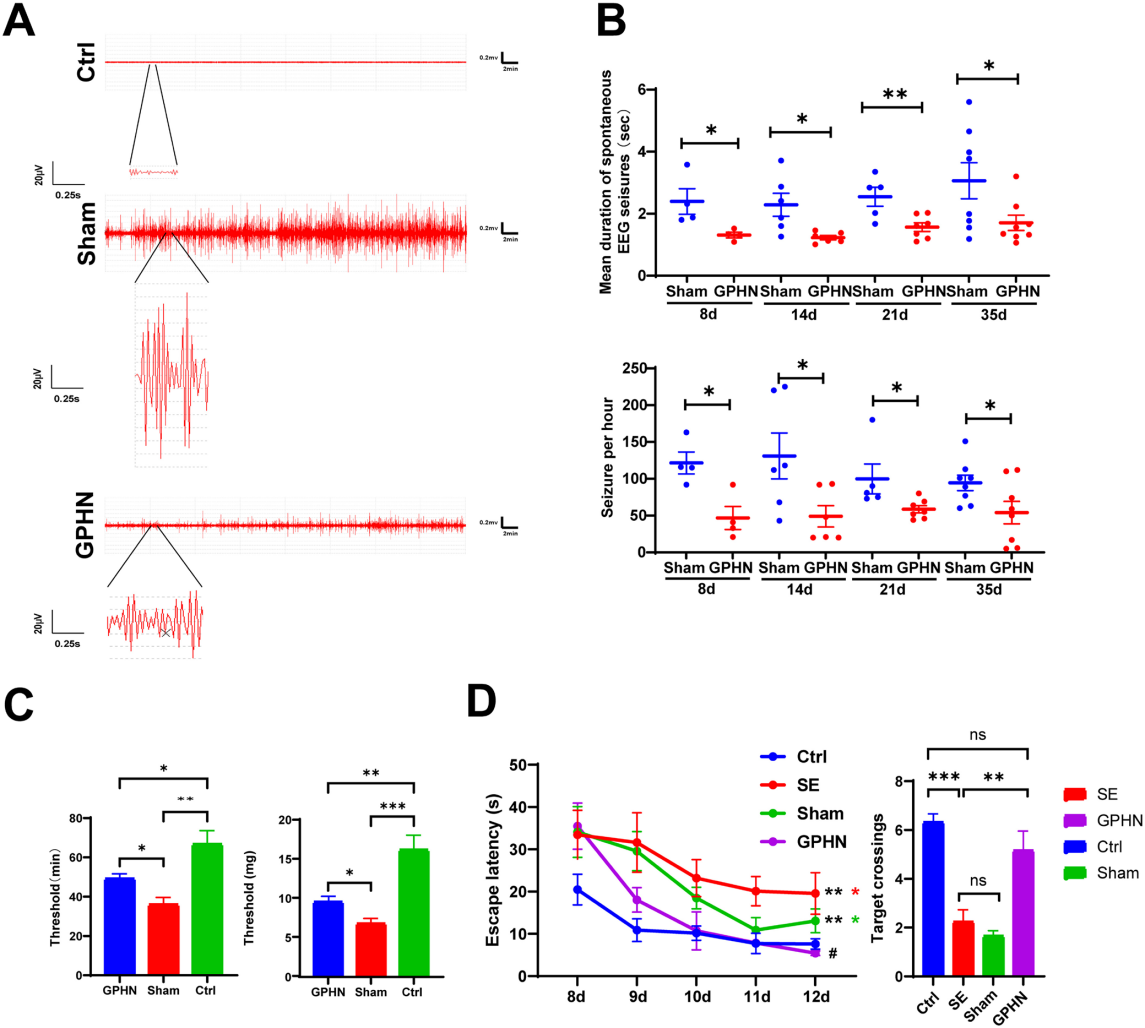
lncRNA‐GPHN ameliorates epileptic discharges and cognitive impairment in SE rats. (A) EEG recordings of spontaneous seizures in control, sham, and lncRNA‐GPHN overexpression groups. Results showed that the frequency and duration of seizures in the lncRNA‐GPHN overexpression group were significantly lower than those in the sham group. (B) The top panel shows the mean duration of spontaneous EEG seizures per hour for each rat (AAV lncRNA‐GPHN injection on the 8th, 14th, 21st, and 35th day; Sham *n* = 4 rats on the 8th day, GPHN *n* = 4 rats on the 8th day; Sham *n* = 6 rats on the 14th day, GPHN *n* = 6 rats on the 14th day; Sham *n* = 5 rats on the 21st day, GPHN *n* = 7 rats on the 21st day; Sham *n* = 8 rats on the 35th day, GPHN *n* = 8 rats on the 35th day; *p < 0.05, **p < 0.01). The bottom panel shows the number of seizures per hour for each rat (AAV lncRNA‐GPHN injection on the 8th, 14th, 21st, and 35th day; Sham *n* = 4 rats on the 8th day, GPHN *n* = 4 rats on the 8th day; Sham *n* = 6 rats on the 14th day, GPHN *n* = 6 rats on the 14th day; Sham *n* = 5 rats on the 21st day, GPHN *n* = 7 rats on the 21st day; Sham *n* = 8 rats on the 35th day, GPHN *n* = 8 rats on the 35th day; *p < 0.05). (C) Latency (left) and dosage (right) required to elicit the first postural clonus in control, sham, and lncRNA‐GPHN overexpression groups using PTZ‐induced seizures. Results showed that the seizure threshold in the lncRNA‐GPHN overexpression group was significantly higher than in the sham group (*n* = 4; *p < 0.05, **p < 0.01, ***p < 0.001). (D) The left panel shows the escape latency during training in the lncRNA‐GPHN overexpression group, which was significantly shorter than in the SE and sham groups (*n* = 6; *p < 0.05, **p < 0.01). The right panel shows the number of platform crossings during the probe trial, with the lncRNA‐GPHN group significantly exceeding the SE and sham groups (*n* = 6; **p < 0.01, ***p < 0.001).

To investigate the role of lncRNA‐GPHN in SE, immature rats were divided into four groups: Ctrl, SE, sham, and lncRNA‐GPHN overexpression groups. Following the establishment of the SE rat model, the sham and lncRNA‐GPHN overexpression groups were intracerebroventricularly injected with AAV2‐empty virus and AAV2‐lncRNA‐GPHN, respectively, 24 h after SE induction. EEG was used to record the epileptic discharges in the Ctrl, sham, and lncRNA‐GPHN overexpression groups. The results showed that the EEG of the lncRNA‐GPHN overexpression group displayed a frequency and duration of epileptic discharges higher than the normal levels of the Ctrl group but significantly lower than the sham group (Figure 2A). The spontaneous seizure duration and hourly seizure frequency of the sham and lncRNA‐GPHN overexpression groups differed significantly at various time points (1, 7, 14, and 28 days), with the lncRNA‐GPHN overexpression group showing significantly shorter durations and lower frequencies at all time points compared to the sham group (*p* < 0.05) (Figure 2B). Furthermore, in the PTZ seizure susceptibility test, the time and dose required to elicit the first postural clonus in the lncRNA‐GPHN overexpression group were significantly higher than in the sham group (*p* < 0.05) (Figure 2C). These results indicate that lncRNA‐GPHN effectively reduces seizure frequency and duration and increases the seizure threshold in SE rats.

Moreover, the MWM test results showed that the escape latency during training in the lncRNA‐GPHN overexpression group was significantly shorter than that in the SE and sham groups (*p* < 0.01), indicating improved learning ability (Figure 2D left). In the probe test, the lncRNA‐GPHN group had significantly more platform crossings than the SE and sham groups (*p* < 0.05), indicating improved memory ability (Figure 2D right). These results suggest that lncRNA‐GPHN significantly improves learning and cognitive impairments in SE rats.

### 3.3 lncRNA‐GPHN Reduces Hippocampal Neuron Death Induced by SE

**FIGURE 3:**
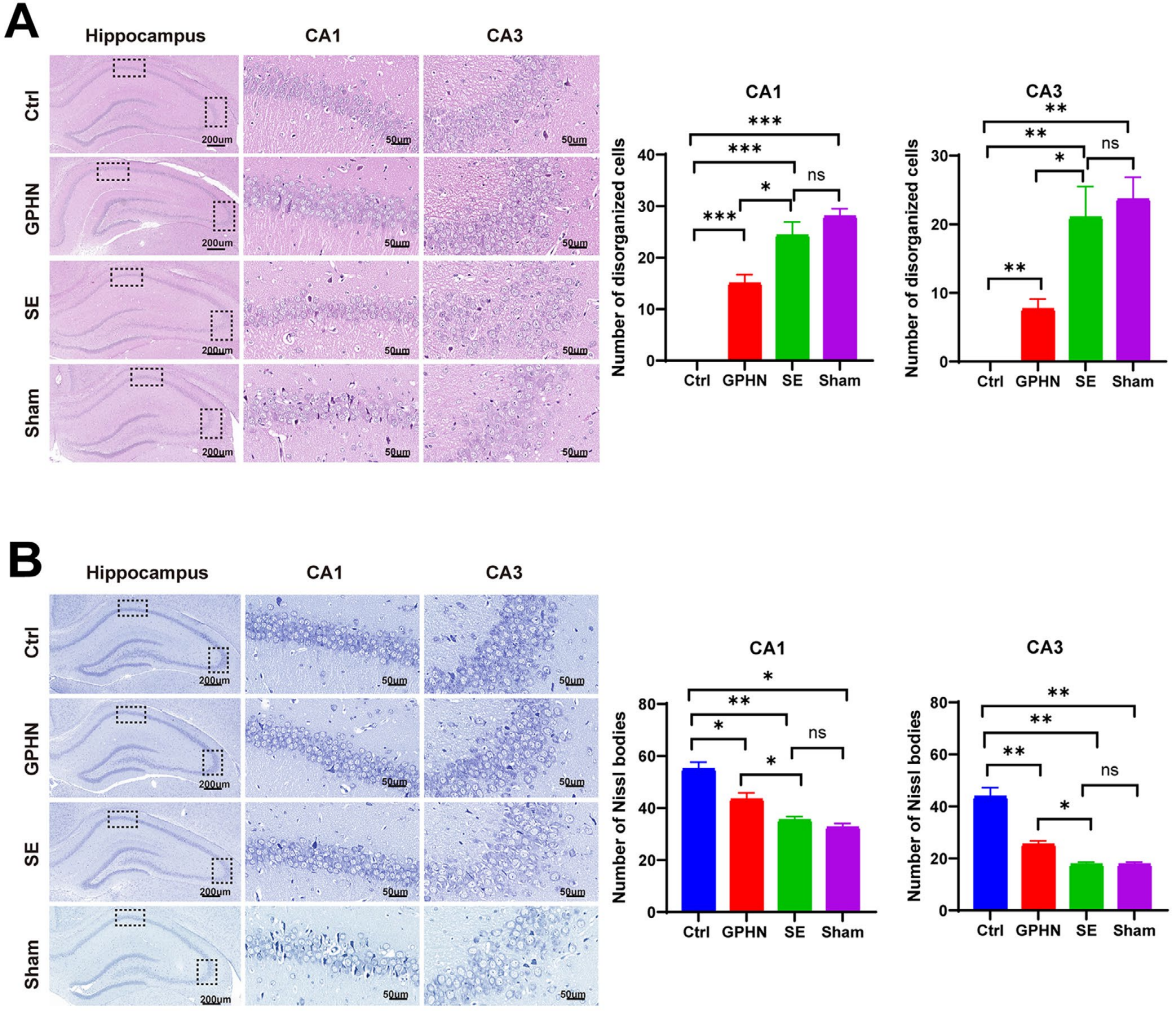
lncRNA‐GPHN alleviated SE‐induced hippocampal neuron death. (A) HE staining was used to observe histological changes in the hippocampus of control, lncRNA‐GPHN overexpression, SE, and sham groups. Results showed that the hippocampal pyramidal cells (including CA1 and CA3 regions) in the lncRNA‐GPHN overexpression group had intact morphological structure, orderly arrangement, clear nucleoli, and evenly distributed chromatin, whereas the SE and sham groups had relatively fewer, swollen, disordered cells with hollow nuclei and ruptured membranes (*n* = 3; *p < 0.05, **p < 0.001, ***p < 0.0001). (B) Nissl staining was used to observe the number of surviving neurons in the hippocampus of each group. Results showed that the number of Nissl bodies in the CA1 and CA3 regions of the lncRNA‐GPHN overexpression group was significantly higher than in the SE and sham groups (*n* = 3; *p < 0.05, **p < 0.001).

To study the impact of lncRNA‐GPHN on SE‐induced neuronal damage in the hippocampus, HE and Nissl staining were performed 7 days after AAV injection. HE staining results showed that the pyramidal cells (including CA1 and CA3 regions) in the Ctrl group's hippocampus had intact morphological structure, orderly arrangement, clear nucleoli, and evenly distributed chromatin. In the SE and sham groups, pyramidal cells were relatively fewer, swollen, and disordered, with hollow nuclei and ruptured membranes. The number of disordered cells in the hippocampal region of SE rats overexpressing lncRNA‐GPHN was significantly reduced compared to the SE and sham groups (*p* < 0.05) (Figure 3A). Nissl staining showed a similar trend, with more and intact pyramidal cells, normal nuclei, and abundant Nissl bodies in the Ctrl group. The SE and sham groups had fewer and damaged pyramidal cells, with swelling, ruptured membranes, disordered arrangement, and significantly reduced Nissl bodies. The number of Nissl bodies in the hippocampal region of lncRNA‐GPHN overexpressing SE rats was significantly higher than in the SE and sham groups (*p* < 0.05) (Figure 3B). These results indicate that lncRNA‐GPHN overexpression significantly reduces SE‐induced hippocampal neuronal damage.

### 3.4 lncRNA‐GPHN Improves SE by Reducing Apoptosis in Hippocampal Neurons

**FIGURE 4:**
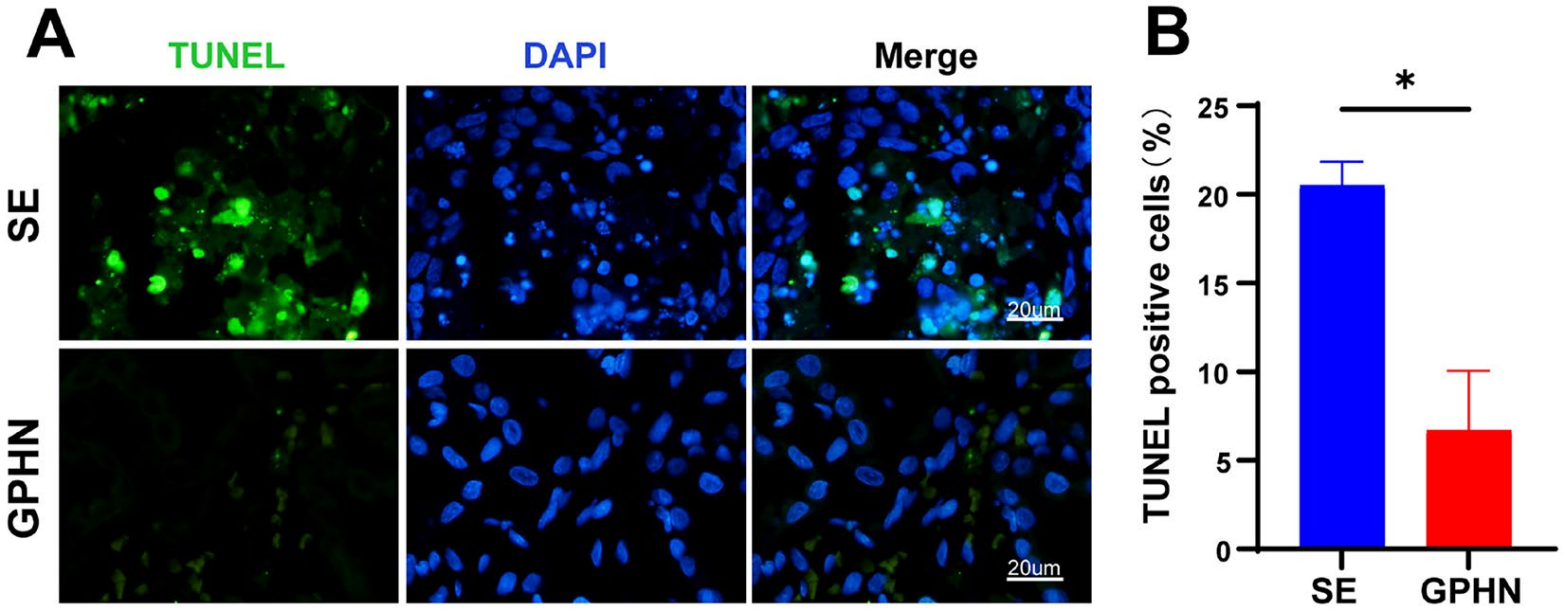
lncRNA‐GPHN ameliorated SE by decreasing apoptosis in hippocampal neurons. (A) TUNEL staining was used to detect the number of apoptotic cells in the hippocampus of each group. Green fluorescence represents TUNEL‐positive cells, and blue fluorescence represents DAPI‐stained nuclei. Results showed that the number of apoptotic cells in the SE group was significantly increased, while the number of apoptotic cells in the lncRNA‐GPHN overexpression group was significantly decreased (*n* = 8; *p < 0.05). (B) Quantitative analysis of TUNEL‐positive cells showed that the proportion of TUNEL‐positive cells in the lncRNA‐GPHN overexpression group was significantly lower than in the SE group (*n* = 8; *p < 0.05).

To investigate the potential regulatory mechanisms of lncRNA‐GPHN in epilepsy, we examined its effect on neuronal apoptosis, as apoptosis was known to play a critical role in neuronal damage associated with epilepsy. TUNEL staining was used to detect the number of apoptotic cells. In TUNEL staining, green fluorescence represents TUNEL‐positive cells, and blue fluorescence represents DAPI‐stained nuclei. The results showed that the SE group had more apoptotic cells, while the lncRNA‐GPHN overexpression group had fewer apoptotic cells (Figure 4A). Quantitative analysis revealed that the proportion of TUNEL‐positive cells in the lncRNA‐GPHN overexpression group was significantly lower than in the SE group (*p* < 0.05) (Figure 4B). These data suggest that lncRNA‐GPHN improves SE‐induced neuronal damage and seizures by inhibiting hippocampal neuronal apoptosis.

### 3.5 Anti‐Epileptic and Anti‐Apoptotic Effects of lncRNA‐GPHN In Vitro

**FIGURE 5:**
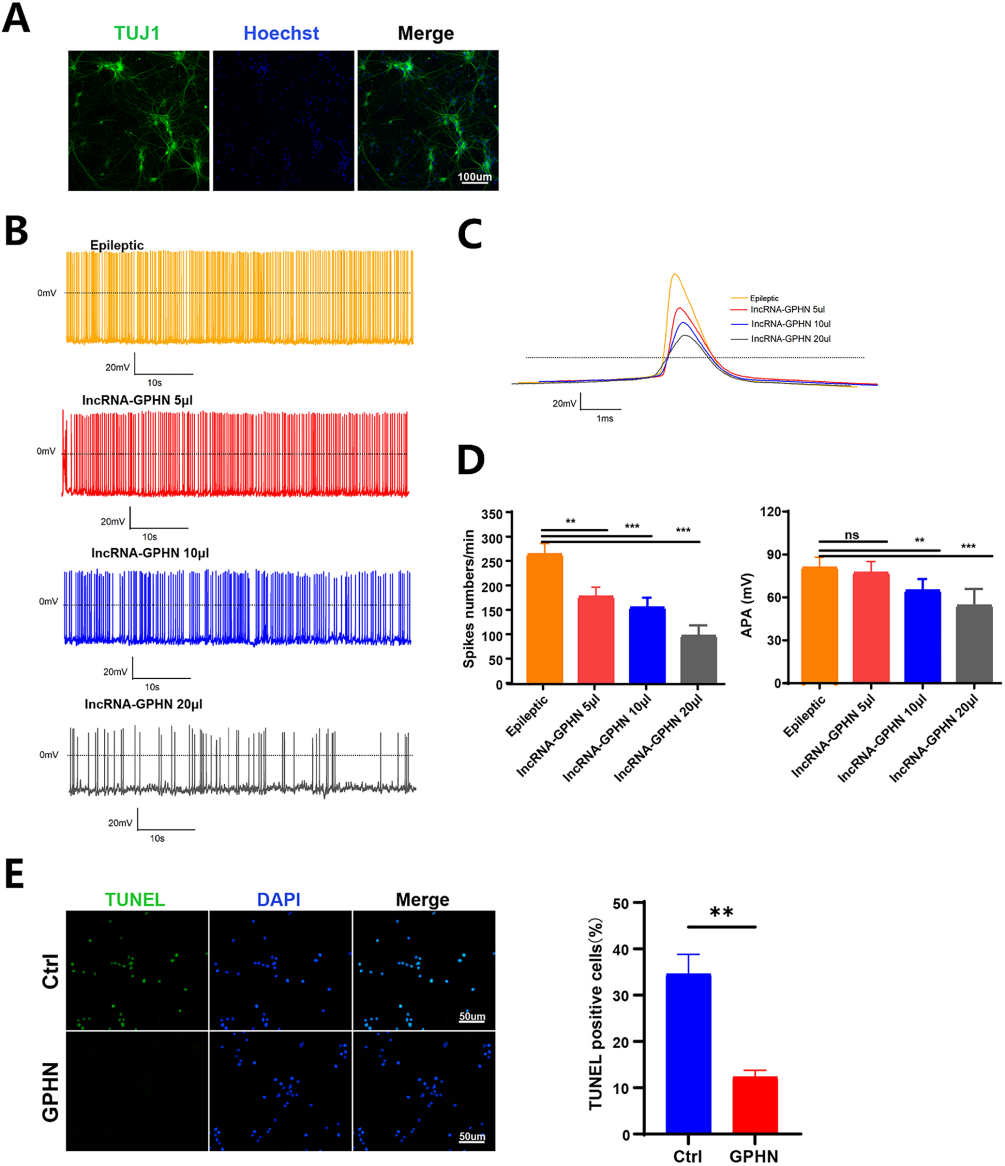
lncRNA‐GPHN exhibited anti‐epileptic and anti‐apoptosis effects in vitro. (A) Immunofluorescence staining was used to identify cells isolated from rat hippocampal tissue. TUJ1 staining shows the neuronal cytoplasm in green, and Hoechst staining shows the nuclei in blue, confirming that the isolated cells are neurons. (B, C) Whole‐cell patch‐clamp recordings were used to monitor the frequency (B) and amplitude (C) of epileptiform discharges in the epileptic cell model after treatment with different concentrations of lncRNA‐GPHN (5 μL, 10 μL, and 20 μL). (D)Results showed that low concentration (5 μL) lncRNA‐GPHN treatment significantly reduced the frequency of action potentials in neuronal cells compared to the control group, while high concentration (20 μL) treatment significantly reduced both the frequency and amplitude of action potentials (*n* = 7; **p < 0.01; ***p < 0.001). (E) TUNEL staining results showed that the number of apoptotic cells was significantly reduced in the lncRNA‐GPHN treatment group compared to the control group. Quantitative analysis showed that the proportion of TUNEL‐positive cells in the lncRNA‐GPHN treatment group was significantly lower than in the control group (*n* = 7; **p < 0.01).

To investigate the antiepileptic effects of lncRNA‐GPHN in vitro, an epileptic cell model of primary hippocampal neurons was constructed. Immunofluorescence staining identified cells isolated from rat hippocampal tissue, with TUJ1 staining the neuronal cytoplasm green and Hoechst staining the nuclei blue, confirming that the isolated cells were neurons (Figure 5A). Whole‐cell patch‐clamp techniques recorded neuronal cell discharges in the epileptic cell model after treatment with different concentrations of lncRNA‐GPHN (5, 10, and 20 μL), dividing the cells into four groups: control, low concentration (5 μL), medium concentration (10 μL), and high concentration (20 μL). Compared to the control group, low concentration (5 μL) lncRNA‐GPHN treatment significantly reduced the frequency of action potentials in neuronal cells (*p* < 0.05), while high concentration (20 μL) treatment more significantly reduced both the frequency and amplitude of action potentials (*p* < 0.01), indicating that lncRNA‐GPHN dose‐dependently reduces neuronal excitability (Figure 5B–D). In TUNEL staining, green fluorescence represents TUNEL‐positive cells, and blue fluorescence represents DAPI‐stained nuclei. The control group had more apoptotic cells, while the lncRNA‐GPHN treatment group had fewer apoptotic cells. Quantitative analysis showed that the proportion of TUNEL‐positive cells in the lncRNA‐GPHN treatment group was significantly lower than in the control group (*p* < 0.05), indicating an anti‐apoptotic effect of lncRNA‐GPHN (Figure 5E). These results suggest that lncRNA‐GPHN dose‐dependently reduces neuronal excitability and apoptosis, exerting its antiepileptic effect in vitro.

### 3.6 lncRNA‐GPHN Regulates Epilepsy by Targeting miR‐320

**FIGURE 6:**
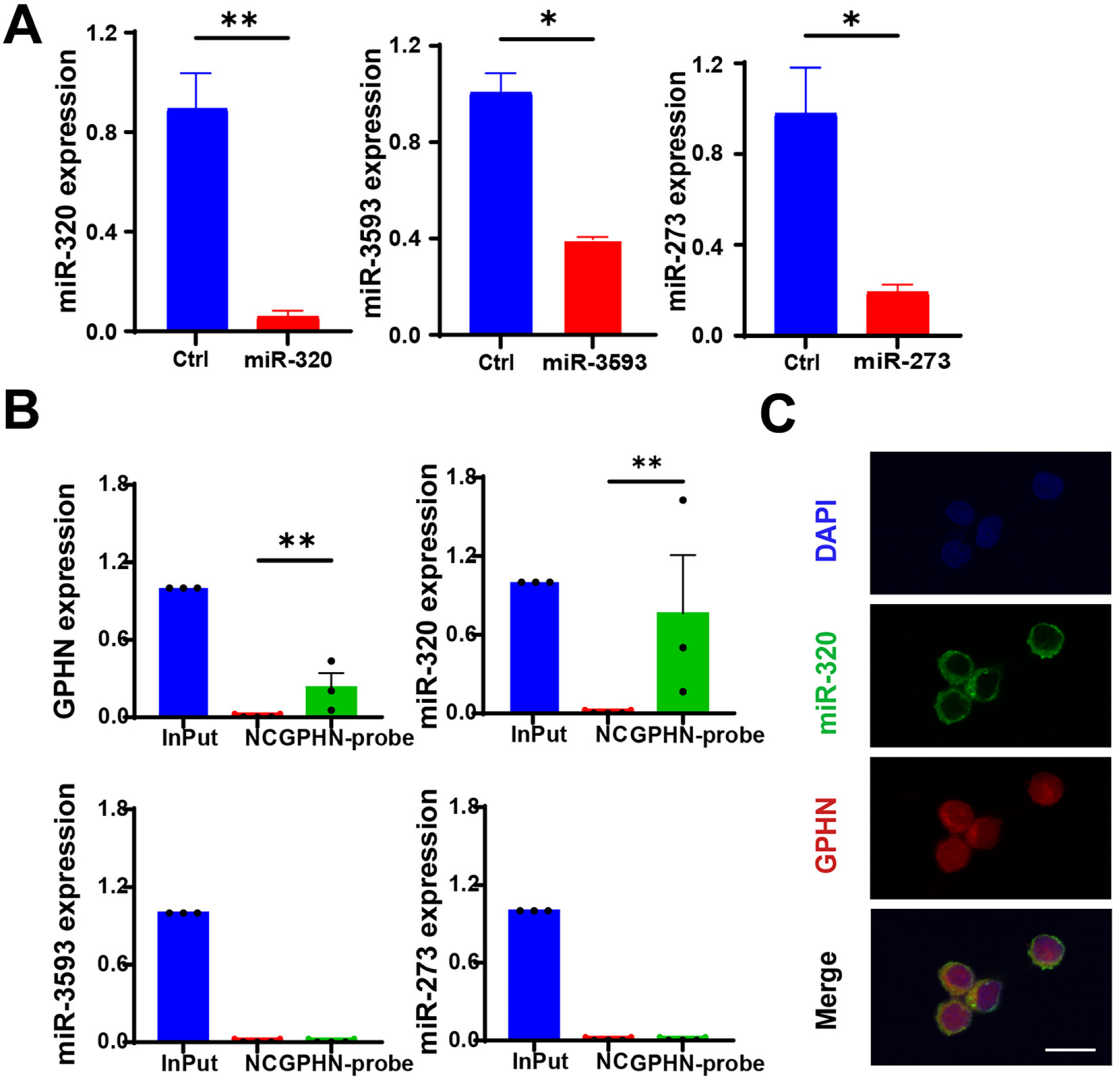
lncRNA‐GPHN directly bonds to miR‐320. (A) qPCR was used to detect the expression levels of miR‐320, miR‐3593, and miR‐273 in neuronal cells after lncRNA‐GPHN overexpression. Results showed that lncRNA‐GPHN overexpression significantly downregulated miR‐320 expression (*n* = 3; *p < 0.05; **p < 0.01). (B) ChIRP was used to detect the ability of lncRNA‐GPHN to directly bind microRNAs. Results showed that lncRNA‐GPHN overexpression significantly increased the binding of GPHN to miR‐320 (*n* = 3; *p < 0.05; **p < 0.01), while no significant changes were observed in the binding of miR‐273 and miR‐3593. (C) FISH staining was used to detect the colocalization signals of lncRNA‐GPHN (red) and miR‐320 (green) within cells, further supporting that lncRNA‐GPHN directly targets miR‐320.

To investigate the regulatory role of lncRNA‐GPHN on apoptosis, q‐PCR was used to detect changes in the expression levels of apoptosis‐related microRNAs (miR‐320, miR‐3593, and miR‐273) after lncRNA‐GPHN overexpression. q‐PCR results showed that miR‐320 expression significantly decreased in lncRNA‐GPHN overexpressing neuronal cells (*p* < 0.01), whereas changes in miR‐273 and miR‐3593 were less significant (*p* < 0.05) (Figure 6A). These results suggest that lncRNA‐GPHN has the most specific regulation of miR‐320.

To confirm whether lncRNA‐GPHN specifically binds to miR‐320 for regulation, ChIRP and immunofluorescence co‐staining were performed. ChIRP results showed a significant increase in the binding of GPHN and miR‐320 in lncRNA‐GPHN overexpressing cells (*p* < 0.01), while no significant changes were observed in the binding of miR‐273 and miR‐3593 (Figure 6B). Immunofluorescence co‐staining showed clear colocalization signals of lncRNA‐GPHN (red) and miR‐320 (green) within cells, further supporting that lncRNA‐GPHN directly targets miR‐320 (Figure 6C). These results suggest that lncRNA‐GPHN regulates apoptosis by targeting miR‐320.

### 3.7 lncRNA‐GPHN Regulates Epilepsy Through the lncRNA‐GPHN/miR‐320/YWHAH Axis

**FIGURE 7:**
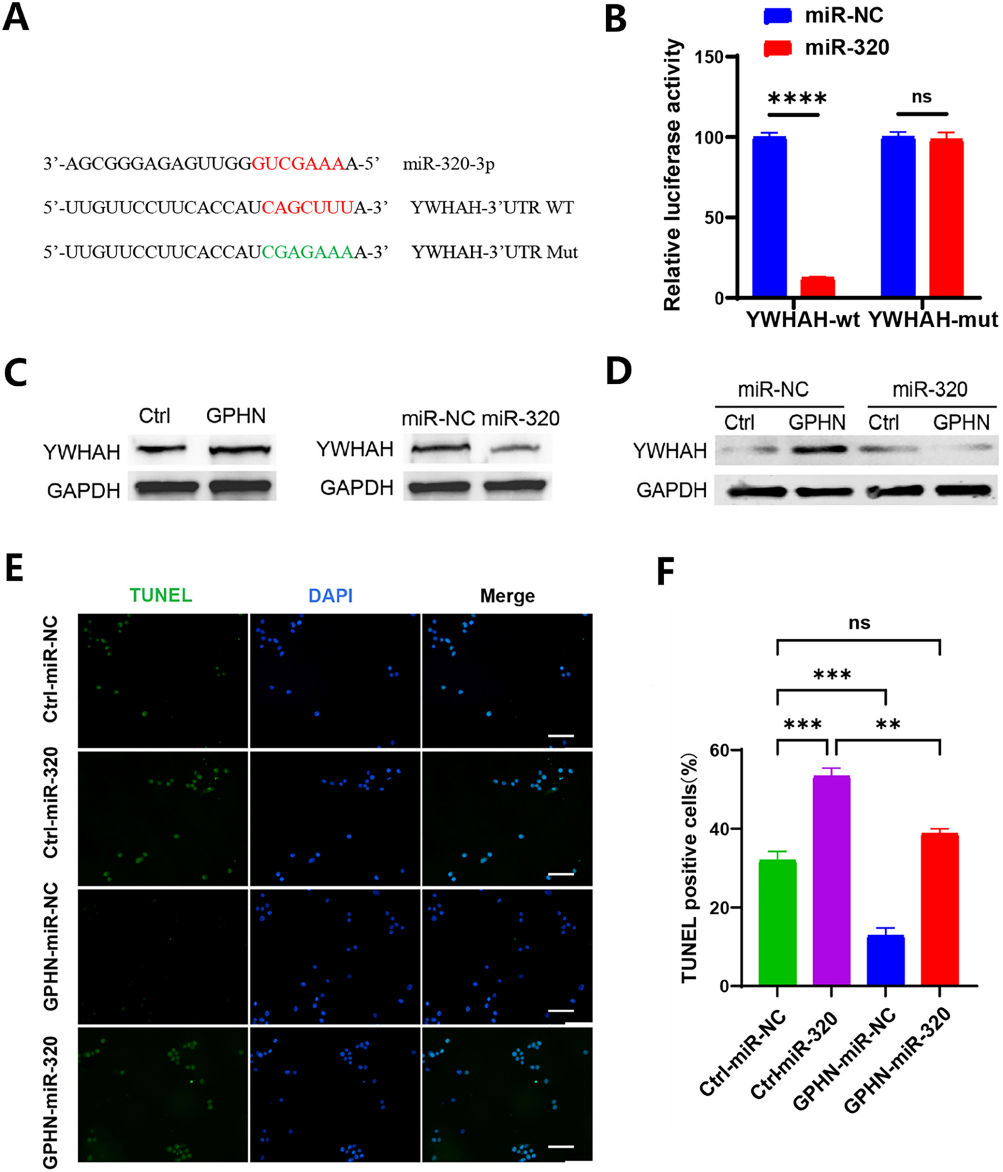
lncRNA‐GPHN regulates apoptosis in epileptic cells by regulating miR‐320 expression. (A) Complementarity between miR‐320‐3p seed sequence and the 3′‐UTR position of YWHAH of rat predicted by a computational and bioinformatics‐based approach using TargetScan. Mutant YWHAH (YWHAH‐3′UTR Mut) was generated by mutating the putative miR‐320‐3p‐binding site (red). Watson–Crick complementarity was marked by red colour. (B) Dual‐luciferase reporter assay was used to detect the binding of miR‐320 to YWHAH‐3′UTR (WT or Mut). Results showed that miR‐320 significantly reduced the luciferase activity of YWHAH‐WT (*n* = 3; ****p < 0.0001), while having no effect on YWHAH‐Mut. (C) Western blot was used to detect YWHAH protein expression levels under different treatment conditions. Results showed that YWHAH protein levels increased after lncRNA‐GPHN overexpression, while miR‐320 overexpression decreased YWHAH protein levels. (D) When both miR‐320 and lncRNA‐GPHN were overexpressed simultaneously, YWHAH expression decreased, indicating that miR‐320 plays a negative regulatory role. (E) TUNEL staining was used to observe apoptosis in different groups (Ctrl+miR‐NC, Ctrl+miR‐320, GPHN+miR‐NC, and GPHN+miR‐320). DAPI (blue) stains the nuclei, and TUNEL (green) represents apoptotic cells. (F) Quantitative analysis of TUNEL‐positive cells showed that the GPHN overexpression group had the lowest number of apoptotic cells, while the Ctrl+miR‐320 group had the highest.

To further explore the downstream targets of lncRNA‐GPHN/miR‐320, we performed bioinformatic analyses that identified several potential targets associated with miR‐320, including the YWHAH protein. YWHAH is closely related to apoptosis and is implicated in the progression of various neurological diseases [18, 19]. Given the crucial role of apoptosis in the pathogenesis of epilepsy, we aimed to explore whether YWHAH is a downstream target regulated by lncRNA‐GPHN/miR‐320. Our analyses identified the YWHAH protein and the binding sites of miR‐320‐3p with the YWHAH‐3'UTR, including wild‐type (WT) and mutant (Mut) sequences (Figure 7A). A dual‐luciferase reporter assay was used to detect the binding of miR‐320 to YWHAH‐3'UTR (WT or Mut). The results showed that miR‐320 significantly reduced the luciferase activity of YWHAH‐WT (*p* < 0.0001), but had no effect on YWHAH‐Mut (Figure 7B), indicating that miR‐320 targets YWHAH, and the binding site becomes ineffective upon mutation, confirming that YWHAH is a downstream target of the lncRNA‐GPHN/miR‐320 axis.

To further clarify the regulatory relationship between lncRNA‐GPHN, miR‐320, and YWHAH, Western blot was used to detect YWHAH protein expression under different treatment conditions. The results showed that YWHAH protein levels increased after lncRNA‐GPHN overexpression, while miR‐320 overexpression decreased YWHAH protein levels (Figure 7C), indicating that GPHN and miR‐320 positively and negatively regulate YWHAH protein expression, respectively. When both miR‐320 and GPHN were overexpressed simultaneously, YWHAH expression decreased, suggesting that miR‐320 plays a negative regulatory role (Figure 7D). These results suggest that lncRNA‐GPHN promotes YWHAH expression by downregulating miR‐320, participating in epilepsy regulation through the lncRNA‐GPHN/miR‐320/YWHAH axis.

To further clarify the role of the lncRNA‐GPHN/miR‐320/YWHAH axis in apoptosis regulation, the experiment was divided into four groups: Ctrl+miR‐NC, Ctrl+miR‐320, GPHN+miR‐NC, and GPHN+miR‐320. TUNEL staining was used to observe apoptosis in each group, with DAPI (blue) staining the nuclei and TUNEL (green) representing apoptotic cells (Figure 7E). The results showed the highest number of apoptotic cells in the Ctrl+miR‐320 group and the fewest in the GPHN+miR‐NC group, indicating that lncRNA‐GPHN overexpression reduces apoptosis, while miR‐320 overexpression increases apoptosis. When both miR‐320 and lncRNA‐GPHN were overexpressed, the number of apoptotic cells partially recovered but remained lower than that in the Ctrl+miR‐320 group (*p* < 0.01), indicating the significant anti‐apoptotic effect of GPHN and its negative regulation of miR‐320 (Figure 7F). Combining the previous research results, we conclude that lncRNA‐GPHN negatively regulates miR‐320 to promote YWHAH expression, reducing neuronal apoptosis, and ultimately decreasing seizures, alleviating hippocampal damage, and improving cognitive impairments.

## 4 Discussion

Recent molecular biology studies have increasingly demonstrated the regulatory roles of lncRNAs in epilepsy. For instance, Rajman et al. discovered that silencing miRNA‐129‐5p prevents synaptic shrinkage and reduces seizure severity [20]. Zayed et al. investigated the expression levels of lncRNAs BDNF‐AS and 17A in the serum of epilepsy patients and explored their correlation with brain‐derived neurotrophic factor (BDNF), phosphorylated cAMP response element‐binding protein (p‐CREB), γ‐aminobutyric acid (GABA), and glutamate, suggesting that these lncRNAs might contribute to epilepsy pathogenesis by inhibiting the GABA/p‐CREB/BDNF pathway and could serve as diagnostic markers [6]. Additionally, lncRNA‐Evf2, which regulates transcription factors crucial for the development of GABAergic interneurons, has been implicated in increased susceptibility to severe and frequent seizures when deficient [21]. These studies highlight the critical regulatory roles of lncRNAs in neurological disorders, including epilepsy, through various molecular mechanisms.

In this study, we observed that lncRNA‐GPHN is predominantly localised in the neuronal cytoplasm of brain tissue and that its expression is significantly reduced following seizures, especially within the first 24 h, before gradually returning to baseline levels. This dynamic change suggests that lncRNA‐GPHN may play a critical regulatory role during the early stages of seizures. Overexpression of lncRNA‐GPHN in SE rats significantly reduced seizure frequency and duration, improved cognitive function, and decreased hippocampal neuron damage and apoptosis. Functional experiments in vitro further demonstrated that lncRNA‐GPHN treatment dose‐dependently reduced neuronal excitability, with low concentrations (5 μL) significantly reducing action potential frequency and high concentrations (20 μL) more significantly reducing both frequency and amplitude. These results indicate that overexpression of lncRNA‐GPHN dose‐dependently reduces neuronal excitability and hippocampal neuron death, thereby mitigating epileptic seizures, neuronal damage, and apoptosis.

How does lncRNA‐GPHN regulate miRNA to reduce epileptic seizures? The competing endogenous RNA (ceRNA) hypothesis suggests that lncRNAs can act as molecular sponges for miRNAs, thereby regulating the expression of target genes [22]. For example, after ischemic stroke, OGD (oxygen–glucose deprivation) induces upregulation of lncRNA H19, which competes for binding with miR‐423‐5p to regulate NOD‐like receptor protein 3 (NLRP3), promoting inflammatory response and inhibiting neurogenesis [23]. Huang et al. also emphasised the importance of lncRNA acting as ceRNA in AD by competitively binding with miRNAs [24]. Our study found that lncRNA‐GPHN targets miR‐320, and overexpression of lncRNA‐GPHN significantly downregulated miR‐320 expression, suggesting that lncRNA‐GPHN primarily participates in the regulation of epilepsy pathogenesis by targeting miR‐320. However, whether it functions as ceRNA in miR‐320 regulation requires further investigation.

miR‐320 plays significant roles in various neurological diseases, often by regulating apoptosis and inflammatory responses involved in brain injury and neurodegenerative diseases [25]. To further explore the roles of lncRNA‐GPHN and miR‐320 in epilepsy pathogenesis, we examined their effects on the expression of the apoptosis‐related protein YWHAH. YWHAH, a member of the 14‐3‐3 protein family, is highly expressed in the brain and is involved in multiple cellular processes, including cell signal transduction, cell cycle control, and apoptosis [26]. The 14‐3‐3 proteins, including YWHAH, influence numerous aspects of brain function, such as neuronal signalling, development, and neuroprotection, with their expression changes potentially impacting neuronal survival and function, thereby contributing to epilepsy pathogenesis [27]. Our study revealed that miR‐320 overexpression significantly reduced YWHAH protein levels, while lncRNA‐GPHN overexpression significantly increased YWHAH expression levels. Combined with the previous finding that lncRNA‐GPHN targets miR‐320 and reduces its expression, we propose the lncRNA‐GPHN/miR‐320/YWHAH regulatory axis: lncRNA‐GPHN negatively regulates miR‐320 to promote the expression of the apoptosis‐related protein YWHAH, reducing neuronal excitability and apoptosis, ultimately alleviating epileptic seizures. This regulatory axis may play a crucial role in epilepsy pathogenesis.

AAV vectors typically require approximately 2 weeks for complete gene expression. Interestingly, our results indicate that as early as 1 week post‐injection, AAV‐mediated GPHN overexpression can significantly reduce epileptic discharges. Similarly, Li et al. [28] observed a significant increase in KLF7 mRNA and protein levels just 1 day after AAV vector injection, a finding validated in both in vitro and in vivo experiments. Importantly, this early expression is sufficient to significantly reduce neuronal damage, decrease apoptosis markers, and protect hippocampal neurons, even before the complete transcription and translation processes are achieved. This suggests that early partial expression mediated by AAV can induce substantial biological effects. Based on the findings from this study and related mechanism research, we hypothesise that similar mechanisms may be involved in our AAV‐mediated GPHN overexpression: (1) Biological effects of early partial expression: Shortly after AAV transduction, sufficient levels of GPHN expression may be produced to exert its biological effects. In critical pathological areas such as the hippocampus, early GPHN expression may rapidly modulate neuronal excitability and suppress excessive discharges [29]. (2) Rapid activation of neuroprotective mechanisms: As observed in the KLF7 study, AAV vectors can immediately activate neuroprotective pathways post‐transduction, enhancing neuronal survival through signalling cascades such as JAK/STAT. These early signal transduction changes may also underlie the rapid effects of GPHN in the epilepsy model. (3) Rapid modulation of synaptic function: Localised overexpression of GPHN may quickly influence synaptic function in these neurons, leading to a suppression of epileptic discharges in a relatively short time [30]. Additionally, GPHN's interactions with neurotransmitter receptors may contribute to immediate changes in synaptic dynamics [31]. We will further explore and elucidate these potential molecular mechanisms in future research.

The findings of this study elucidate the pivotal role of lncRNA‐GPHN in the pathogenesis of epilepsy, offering new insights into its treatment. As a novel molecular target, lncRNA‐GPHN could be modulated to influence the course of epilepsy, potentially through antisense oligonucleotide (ASO) technology, providing opportunities for the development of new anti‐epileptic drugs [32]. Additionally, CRISPR/Cas9‐mediated gene knockout technology could be employed to specifically target lncRNA‐GPHN for precise epilepsy treatment [33]. Future research should further explore the application of these technologies in epilepsy therapy to develop more effective treatment strategies.

However, this study has limitations. First, while we observed temporal changes in endogenous lncRNA‐GPHN expression following status epilepticus (SE), the experimental design did not directly measure the dynamic expression of exogenous AAV‐mediated lncRNA‐GPHN at different time points. This gap limits our ability to analyse the temporal relationship between exogenous lncRNA‐GPHN concentration and its anti‐epileptic efficacy. Further validation of lncRNA‐GPHN, miR‐320, and YWHAH expression and function in clinical samples is necessary, especially considering the complex and multifactorial nature of epilepsy. Given that lncRNA‐GPHN may be one of many regulatory factors involved, further exploration of its interactions with other molecules and signalling pathways is warranted [34]. Secondly, electrophysiological analyses were confined to SE‐induced hyperexcitable neurons, without investigating the effects of lncRNA‐GPHN in normal neurons. This omission hinders our understanding of whether the inhibitory effects of lncRNA‐GPHN are specific to pathological conditions or if they also apply to the regulation of neuronal excitability in general. If the latter holds true, it may raise concerns about potential side effects under normal conditions. Additionally, this study primarily focused on male mice, but research indicates that sex differences significantly impact neurological conditions, including epilepsy [35, 36]. Variations in sex hormones and chromosomes may alter lncRNA expression, with evidence suggesting that females, especially under oestrogen's influence, have enhanced neuroprotective mechanisms [37, 38]. Such sex‐based differences in blood–brain barrier permeability, neuroinflammatory pathways, and neuroactive steroids could affect seizure susceptibility and severity [36, 39]. Future studies should examine lncRNA‐GPHN expression and function in female mice, including hormonal influences. This research may reveal sex‐specific regulatory pathways and clarify lncRNA‐GPHN's role in female neuroprotection and apoptosis, supporting individualised, sex‐specific epilepsy therapies.

## 5 Conclusion

Our study, through the construction of an SE rat model and in vitro cell model, reveals the significant role and regulatory mechanism of lncRNA‐GPHN in epilepsy pathogenesis via the miR‐320/YWHAH regulatory axis. This discovery provides new perspectives for understanding the mechanisms underlying epilepsy and offers potential targets for developing new antiepileptic therapeutic strategies. Future research should further validate the applicability of this regulatory axis in clinical samples to bring new therapeutic hope to epilepsy patients.

## Author Contributions

**Jun Chen:** data curation (lead), formal analysis (lead), investigation (lead), validation (lead), visualization (lead), writing – original draft (lead). **Xueyi Rao:** data curation (lead), formal analysis (lead), investigation (lead), visualization (lead), writing – original draft (lead). **Xiaoqian Wang:** data curation (equal), formal analysis (equal), investigation (equal), software (lead). **Yang Li:** data curation (equal), formal analysis (equal), investigation (equal), resources (equal), software (equal). **Yajun Shen:** conceptualization (lead), methodology (lead), project administration (lead), supervision (lead), writing – review and editing (lead). **Jing Gan:** conceptualization (lead), funding acquisition (lead), investigation (lead), methodology (lead), project administration (lead), supervision (lead), writing – review and editing (lead).

## Ethics Statement

All experimental procedures were approved by the Institutional Animal Care and Use Committees of Sichuan University.

## Consent

The authors have nothing to report.

## Conflicts of Interest

The authors declare that they have no potential conflicts of interest. Jun Chen and Xueyi Rao contributed equally to this work.

## Data Availability

The data that support the findings of this study are available from the corresponding author upon reasonable request.
